# Nurses’ compliance with infection control, safety measures, communication, and protocol adherence in operating rooms of governmental hospitals in the Gaza strip

**DOI:** 10.3389/frhs.2025.1657817

**Published:** 2025-08-29

**Authors:** Mohammed A. Abu Rahmah, Bothyna B. ELssyed Etewa, Fatma Al’Haj Ahmad, Abdel Hamid El Bilbeisi

**Affiliations:** ^1^ Department of Public Health Nursing, School of Public Health, University of Albutana, Rufaa, Sudan; ^2^ Department of Nutrition, School of Medicine and Health Sciences, University of Palestine, Gaza Strip, Palestine; ^3^ Department of Clinical Nutrition, Faculty of Applied Medical Sciences, Al Azhar University of Gaza, Gaza Strip, Palestine

**Keywords:** Gaza strip, infection control, nurses' compliance, operating rooms, patient safety

## Abstract

**Background:**

This study assessed nurses’ compliance with infection control, safety measures, communication, and protocol adherence in operating rooms of government hospitals in Gaza.

**Methods:**

A cross-sectional study was conducted in 2023 across the three main governmental hospitals in Gaza: Al Shifa Medical Complex, Nasser Medical Complex, and Gaza European Hospital. A census sampling method included 150 nurses working in operating rooms who met inclusion criteria. Data were collected using a structured, validated questionnaire covering demographics and six domains of patient safety based on the WHO Patient Safety Assessment Tool. Statistical analysis was performed using SPSS version 26, with significance set at *p*-value < 0.05.

**Results:**

Among 150 nurses (78% male, mean age 32.94 ± 7.3 years); most (71.4%) held a bachelor’s degree; and 43.3% had specialized operating room training. Positive responses on sterilization and cleaning averaged 46.7%, highest at Al Shifa (51.8%). Key practices such as instrument transport in sealed trolleys (80.7%) and immediate cleaning post-procedure (83.3%) were well reported. Intraoperative precaution compliance was 73.3%, with high rates of proper hand scrubbing (84.7%) but lower adherence to checklist completion (65.3%). Immediate post-operative monitoring adherence was 68.7%, with oxygen saturation measurement (88%) and pain assessment (77.3%) rated highly. Communication satisfaction was 72.7%, though cooperation during patient positioning was only 46%. About 66.7% reported positive views on policies and training, yet 75.3% expressed a need for more patient safety education. A high incidence of adverse events was reported by 93.3%, with reluctance to document errors noted by 68%.

**Conclusion:**

This study highlights moderate compliance with infection control in Gaza Strip operating rooms but reveals significant gaps in protocol adherence, documentation, and error reporting. It highlights the need for better training, resource support, and a non-punitive safety culture. Policymakers and hospital leaders must prioritize these to improve surgical safety and patient care.

## Introduction

Nurses play a vital role in maintaining and enhancing patient safety in the operating room environment (1). Their continuous involvement in perioperative care, including infection control, adherence to safety protocols, monitoring, and communication, directly influences surgical outcomes (2). Studies show that nurses’ perceptions, attitudes, and adherence to safety measures are critical factors that determine the effectiveness of patient safety strategies (3, 4). However, in many resource-limited settings such as the Gaza Strip, challenges including limited training opportunities, inadequate resources, and high workload contribute to gaps in safety practices and reporting (5).

The Gaza Strip, a densely populated region affected by prolonged political instability and conflict, faces significant healthcare challenges, including restrictions on supplies, infrastructure damage, and workforce shortages (6). These conditions exacerbate risks in surgical care, making it imperative to understand the current status of patient safety from the perspective of frontline healthcare providers, especially nurses (7). Despite international efforts to improve surgical safety through standardized protocols such as the World Health Organization (WHO) Surgical Safety Checklist, implementation and adherence vary significantly across different healthcare settings (8). Recent studies emphasize the importance of comprehensive assessments of patient safety culture and practices within operating rooms to identify weaknesses and opportunities for intervention (9, 10). For example, a 2024 study in Jordan revealed that nurses’ knowledge and attitudes toward infection control and safety protocols strongly correlated with reduced surgical site infections and improved teamwork (11). Similarly, research in Egypt highlighted the impact of communication and teamwork training on reducing adverse events in perioperative care (12).

To the best of our knowledge, limited data exist on the specific experiences and perceptions of nurses working in the Gaza Strip's government hospitals, particularly regarding infection control, safety measures, communication, adherence to protocols, and the prevalence of adverse events. Addressing this gap is essential for designing targeted interventions to improve patient safety and surgical outcomes in this challenging environment. Actually, there is a pressing need to evaluate and enhance patient safety within the operating rooms of governmental hospitals in the Gaza Strip, a context marked by unique structural, resource, and conflict-related constraints. Understanding nurses’ compliance with safety practices is fundamental, as nurses are frontline caregivers who directly influence perioperative care quality and patient outcomes (13).

Furthermore, the Gaza Strip's healthcare system, under strain from ongoing conflict and resource shortages, exemplifies these challenges (14). Investigating nurses’ viewpoints provides crucial insights into the current state of safety measures, barriers to compliance, and opportunities for improvement within this fragile context. This research provides a timely and necessary evaluation of nurses’ compliance with patient safety in Gaza's governmental operating rooms. The findings will contribute to building evidence-based strategies tailored to the region's specific challenges, ultimately improving surgical care quality and patient outcomes in a context where healthcare resilience is vital.

## Materials and methods

### Study design

This observational, descriptive, and analytical cross-sectional study aims to assess nurses’ perceptions of patient safety in operating rooms at government hospitals in the Gaza Strip. Observation was incorporated by training data collectors to use an observational checklist during the interviews. This checklist allowed them to directly verify certain safety practices and protocol adherence in the operating rooms while interacting with the nurses.

### Study location and period

The study was conducted in 2023, before the outbreak of the Gaza war, in the operating rooms of the three primary government hospitals in the Gaza Strip: Al Shifa Medical Complex, Nasser Medical Complex, and Gaza European Hospital.

### Study population

The study included all nurses, regardless of gender, who were working in the main operating rooms of the selected hospitals in the Gaza Strip, as long as they met the inclusion criteria and were available during the study period. Nurses were excluded if they had less than six months of employment, were volunteers, worked in minor operating rooms, or chose not to participate.

### Sample size and sampling technique

A census sampling method was employed to include all nurses working in the operating rooms of the three selected hospitals who met the inclusion criteria at the time of data collection. Out of 162 eligible nurses, 150 took part in the study, yielding a response rate of 92.6%. The remaining 12 nurses either opted not to participate or were absent during the data collection period.

### Data collection

Data collection was carried out by a team of five trained individuals, including the researcher and four nurses. The researcher provided them with a detailed explanation and training on the study's purpose, objectives, procedures, and guidelines for using the observational checklist to ensure accurate data collection.

### Interview-based questionnaire

Data were gathered from the nurses using a structured, pre-tested, and validated questionnaire. The instrument comprised two main sections: (1) Demographic and professional information of the participants, including age, gender, educational qualifications, specialized training, completed courses, and years of experience; and (2) The World Health Organization's “Patient Safety Assessment Tool,” which was employed to assess patient safety within the operating rooms of government hospitals in the Gaza Strip (15).

The Patient Safety Assessment Tool consisted of six key domains: (1) Sterilization and cleaning, (2) Intraoperative precautionary measures, (3) Immediate post-operative monitoring, (4) Communication, (5) Policies, procedures, protocols, and training, and (6) Adverse events (15).

#### Scoring method

For each domain, the total number of responses, both “Yes” (positive) and “No” (negative), was calculated by summing all answers to the sub-questions within that domain. This total (Yes + No) was then divided by the number of sub-questions in that domain to determine the overall response rate or level of engagement. Following this, the responses were categorized into “Yes” (positive responses) and “No” (negative responses), and this classification was used to examine the response trends within each domain.

Data on the six WHO patient safety domains were collected through structured interviews to ensure clear understanding and better response rates. To reduce response bias, interviewers emphasized confidentiality, used neutral wording, and assured anonymity to encourage honest answers. The questionnaire was also refined through a pilot study to improve clarity. Although self-reporting has limitations, these steps helped minimize bias.

### Pilot study

A pilot study with 15 nurses was carried out at Al Shifa Medical Complex, selected for its sizable operating room nursing team and ease of access. The pilot tested the questionnaire's clarity and practicality, and based on participant feedback, minor adjustments were made to improve comprehension and data accuracy for the main study.

### Data analysis

Statistical analysis was carried out using SPSS version 26. This included defining variables, entering and cleaning the data, followed by analysis. Continuous variables were expressed as means ± standard deviation (SD), and categorical variables were presented as percentages. The Chi-square test was applied to assess differences between categorical variables, and One-Way ANOVA test was used to determine the mean differences in quantitative variables, with a *p*-value below 0.05 indicating statistical significance.

## Results

A total of 150 nurses were included in the final analysis. Among them, 117 (78.0%) were male and 33 (22.0%) were female. The participants’ average age was 32.94 ± 7.3 years. Nearly half of the nurses (48.0%) were employed at Nasser Medical Complex, followed by 37.3% at Al Shifa Medical Complex and 14.7% at Gaza European Hospital. Regarding educational background, 71.4% held a bachelor's degree in nursing, 27.3% had a diploma, and only 1.3% possessed a master's degree. Furthermore, 43.3% had completed specialized training in operating room care, and 41.3% had attended relevant training courses. The average length of experience working in operating rooms was 7.23 ± 6.18 years, with an average weekly workload of 37.4 ± 5.45 h. No statistically significant differences were observed between hospitals (*p*-values > 0.05 for all), as detailed in Table 1.

**Table 1:** Socio-demographic characteristics of the study participants by hospitals.

Variables	Total (*n* = 150)	Al Shifa medical complex (*n* = 56)	Nasser medical complex (*n* = 72)	European Gaza hospital (*n* = 22)	P. value
	No (%)	No (%)	No (%)	No (%)	
Age (years)					
Mean ± SD	32.94 ± 7.3	32.28 ± 6.9	32.87 ± 7.3	34.86 ± 8.2	0.337
Gender					
Males	117 (78.0)	44 (78.6)	57 (79.2)	16 (72.7)	0.809
Females	33 (22.0)	12 (21.4)	15 (20.8)	6.0 (27.3)	
Qualifications					
Diploma	41 (27.3)	17 (30.4)	19 (26.4)	5.0 (22.7)	0.390
Bachelor	107 (71.4)	37 (66.1)	53 (73.6)	17 (77.3)	
Master	2.0 (1.3)	2.0 (3.5)	0.0 (0.0)	0.0 (0.0)	
Special study in operation room care					
Yes	65 (43.3)	29 (51.8)	28 (38.9)	8.0 (36.4)	0.267
No	85 (56.7)	27 (48.2)	44 (61.1)	14 (63.6)	
Training course in operation room care					
Yes	62 (41.3)	28 (50.0)	28 (38.9)	6.0 (27.3)	0.157
No	88 (58.7)	28 (50.0)	44 (61.1)	16 (72.7)	
Total work experiences in operating room (year)					
Mean ± SD	7.23 ± 6.18	6.55 ± 5.4	7.26 ± 6.2	8.86 ± 7.4	0.334
Total hours of work weekly					
Mean ± SD	37.40 ± 5.45	37.51 ± 6.2	37.27 ± 4.8	37.54 ± 5.4	0.962

Continuous variables were expressed as means ± standard deviation (SD), and categorical variables were presented as percentages. The Chi-square test was applied to assess differences between categorical variables, and One-Way ANOVA test was used to determine the mean differences in quantitative variables, with a *p*-value below 0.05 indicating statistical significance.

As shown in Table 2, only 46.7% of nurses across all hospitals gave positive feedback regarding sterilization and cleaning practices, with Al Shifa Medical Complex reporting the highest compliance (51.8%), followed by Nasser Medical Complex (44.4%), and Gaza European Hospital with the lowest (40.9%). However, these differences were not statistically significant (*p*-value = 0.599). A majority of nurses indicated adherence to key infection control practices: 80.7% reported that used instruments were transported in a sealed trolley to the washing area, 83.3% confirmed that the operation room was cleaned immediately after procedures, and 77.3% stated that biomedical waste was appropriately segregated. Additionally, 76.7% mentioned surface cleaning occurred when the operating room was idle, and 84.7% noted that the operative field was properly draped by the surgeon.

**Table 2:** Responses of the study participants for sterilizing and cleaning by hospitals.

Variables	Total (*n* = 150)	Al Shifa medical complex (*n* = 56)	Nasser medical complex (*n* = 72)	European Gaza hospital (*n* = 22)	P. value
	No (%)	No (%)	No (%)	No (%)	
Sterilizing and cleaning					
1. Used instruments are moved in a sealed trolley and taken to the instrument washing area?					
Yes	121 (80.7)	46 (82.1)	57 (79.2)	18 (81.8)	0.904
No	29 (19.3)	10 (17.9)	15 (20.8)	4.0 (18.2)	
2. Cleaning the operation room is done after the case immediately?					
Yes	125 (83.3)	47 (83.9)	60 (83.3)	18 (81.8)	0.975
No	25 (16.7)	9.0 (16.1)	12 (16.7)	4.0 (18.2)	
3. Biomedical waste is appropriately segregated?					
Yes	116 (77.3)	45 (80.4)	55 (76.4)	16 (72.7)	0.743
No	34 (22.7)	11 (19.6)	17 (23.6)	6.0 (27.3)	
4. Surface cleaning is done when there is no case					
Yes	115 (76.7)	41 (73.2)	56 (77.8)	18 (81.8)	0.688
No	35 (23.3)	15 (26.8)	16 (22.2)	4.0 (18.2)	
5. Is the surgeon covered the operative field correctly by sterile sheet?					
Yes	127 (84.7)	49 (87.5)	61 (84.7)	17 (77.3)	0.529
No	23 (15.3)	7.0 (12.5)	11 (15.3)	5.0 (22.7)	
Total of positive responses	**70** **(46.7)**	**29** **(51.8)**	**32** **(44.4)**	**9.0** **(40.9)**	**0** **.** **599**
Total of negative responses	**80** (**53.3)**	**27** (**48.2)**	**40** (**55.6)**	**13** (**59.1)**	

Categorical variables were presented as percentages. The Chi-square test was applied to assess differences between categorical variables, with a *p*-value below 0.05 indicating statistical significance.

The bold values indicate total of positive and negative responses.

Regarding intraoperative precautions, 73.3% of nurses overall reported compliance, with Al Shifa Medical Complex again leading at 80.4%, followed by Nasser Medical Complex (72.2%) and Gaza European Hospital (59.1%), though the differences were not statistically significant (*p*-value = 0.154). Furthermore, 84.7% of nurses stated that hand scrubbing was performed properly (3–5 min), while 72.7% and 76.7% confirmed that the times of skin incision and closure were correctly recorded. Compliance was lower in areas such as reading the third part of the time-out checklist aloud before skin closure (65.3%) and documentation of operative notes by nurses (62.7%). Post-operative care documentation was moderately observed, with 80% reporting it was written by the surgeon, but only 56.7% confirmed it was signed. Additionally, 66% noted proper application of discharge criteria, and 60% believed the staffing levels in the operation department were adequate as shown in Table 3.

**Table 3:** Responses of the study participants for precautions of intra operative period by hospitals.

Variables	Total (*n* = 150)	Al Shifa medical complex (*n* = 56)	Nasser medical complex (*n* = 72)	European Gaza hospital (*n* = 22)	P. value
	No (%)	No (%)	No (%)	No (%)	
Precautions of intra operative period					
1. When doing scrubbing you wash and brush your hands 3–5m?					
Yes	127 (84.7)	46 (82.1)	62 (86.1)	19 (86.4)	0.803
No	23 (15.3)	10 (17.9)	10 (13.9)	3.0 (13.6)	
2. Skin incision time correctly noted down on the white board, billing sheet and master register of operating theatre?					
Yes	109 (72.7)	40 (71.4)	52 (72.2)	17 (77.3)	0.867
No	41 (27.3)	16 (28.6)	20 (27.8)	5.0 (22.7)	
3. Skin closure time written on the board, billing sheet and master register?					
Yes	115 (76.7)	40 (71.4)	56 (77.8)	19 (86.4)	0.356
No	35 (23.3)	16 (28.6)	16 (22.2)	3.0 (13.6)	
4. Third part of time out checklist read out loudly before skin closure?					
Yes	98 (65.3)	35 (62.5)	47 (65.3)	16 (72.7)	0.694
No	52 (34.7)	21 (37.5)	25 (34.7)	6.0 (27.3)	
5. Operative notes written and signed by the nurse/operation room technician?					
Yes	94 (62.7)	35 (62.5)	45 (62.5)	14 (63.6)	0.940
No	56 (37.3)	21 (37.5)	27 (37.5)	8.0 (36.4)	
6. Post-operative plan of care written by the surgeon/assistant?					
Yes	120 (80.0)	46 (82.1)	57 (79.2)	17 (77.3)	0.863
No	30 (20.0)	10 (17.9)	15 (20.8)	5.0 (22.7)	
7. Post-operative care plan signed by surgeon/assistant?					
Yes	85 (56.7)	32 (57.1)	40 (55.6)	13 (59.1)	0.954
No	65 (43.3)	24 (42.9)	32 (44.4)	9.0 (40.9)	
8. Discharge criteria applied to decide while transferring the patient to recovery room, intensive care unit, ward or home?					
Yes	99 (66.0)	37 (66.1)	46 (63.9)	16 (72.7)	0.746
No	51 (34.0)	19 (33.9)	26 (36.1)	6.0 (27.3)	
9. Is the surgical staff enough at operation department?					
Yes	90 (60.0)	34 (60.7)	42 (58.3)	14 (63.6)	0.897
No	60 (40.0)	22 (39.3)	30 (41.7)	8.0 (36.4)	
Total of positive responses	**110** (**73.3)**	**45** (**80.4)**	**52** (**72.2)**	**13** (**59.1)**	**0**.**154**
Total of negative responses	**40** (**26.7)**	**11** (**19.6)**	**20** (**27.8)**	**9.0** (**40.9)**	

Categorical variables were presented as percentages. The Chi-square test was applied to assess differences between categorical variables, with a *p*-value below 0.05 indicating statistical significance.

The bold values indicate total of positive and negative responses.

In terms of immediate post-operative monitoring, 68.7% (*n* = 103) of nurses reported adherence to proper monitoring protocols, with Al Shifa Medical Complex showing the highest compliance (75.0%), followed by Nasser Medical Complex (68.1%) and Gaza European Hospital (54.5%). However, these differences were not statistically significant (*p*-value = 0.213). Specific aspects of monitoring included documentation by the recovery room nurse (72.0%), patient awareness reporting (66.7%), post-operative pain score assessment (77.3%), oxygen saturation measurement (88.0%), observation of nausea and vomiting (78.7%), and detection of post-operative headache (62.0%) as shown in Table 4.

**Table 4:** Responses of the study participants for the immediate post-operative monitoring, and communication by hospitals.

Variables	Total (*n* = 150)	Al Shifa medical complex (*n* = 56)	Nasser medical complex (*n* = 72)	European Gaza hospital (*n* = 22)	P. value
	No (%)	No (%)	No (%)	No (%)	
Immediate post-operative monitoring					
1. Recovery room—monitoring documented by the recovery room nurse					
Yes	108 (72.0)	43 (76.8)	51 (70.8)	14 (63.6)	0.485
No	42 (28.0)	13 (23.2)	21 (29.2)	8.0 (36.4)	
2. Was there any awareness (patient awake) reported/observed?					
Yes	100 (66.7)	36 (64.3)	48 (66.7)	16 (72.7)	0.776
No	50 (33.3)	20 (35.7)	24 (33.3)	6.0 (27.3)	
3. Post-operative pain score measured?					
Yes	116 (77.3)	42 (75.0)	57 (79.2)	17 (77.3)	0.856
No	34 (22.7)	14 (25.0)	15 (20.8)	5.0 (22.7)	
4. Post-operative oxygen saturation measured?					
Yes	132 (88.0)	49 (87.5)	64 (88.9)	19 (86.4)	0.940
No	18 (12.0)	7.0 (12.5)	8.0 (11.1)	3.0 (13.6)	
5. Post-operative nausea and vomiting observed?					
Yes	118 (78.7)	43 (76.8)	57 (79.2)	18 (81.8)	0.879
No	32 (21.3)	13 (23.2)	15 (20.8)	4.0 (18.2)	
6. Post-operative headache observed?					
Yes	93 (62.0)	34 (60.7)	43 (59.7)	16 (72.7)	0.529
No	57 (38.0)	22 (39.3)	29 (40.3)	6.0 (27.3)	
Total of positive responses	**103** (**68.7)**	**42** (**75.0)**	**49** (**68.1)**	**12** (**54.5)**	**0**.**213**
Total of negative responses	**47** (**31.3)**	**14** (**25.0)**	**23** (**31.9)**	**10** (**45.5)**	
Communication					
1. You think the communication between surgical team is excellent?					
Yes	108 (72.0)	43 (76.8)	51 (70.8)	14 (63.6)	0.485
No	42 (28.0)	13 (23.2)	21 (29.2)	8.0 (36.4)	
2. Surgical team exchange information by respect and professional way during surgical operation?					
Yes	111 (74.0)	43 (76.8)	53 (73.6)	15 (68.2)	0.734
No	39 (26.0)	13 (23.2)	19 (26.4)	7.0 (31.8)	
3. Is the surgical team cooperating at positioning the patient on surgical table?					
Yes	69 (46.0)	25 (44.6)	32 (44.4)	12 (54.5)	0.684
No	81 (54.0)	31 (55.4)	40 (55.6)	10 (45.5)	
Total of positive responses	**109** (**72.7)**	**43** (**76.8)**	**53** (**73.6)**	**13** (**59.1)**	**0**.**279**
Total of negative responses	**41** (**27.3)**	**13** (**23.2)**	**19** (**26.4)**	**9.0** (**40.9)**	

Categorical variables were presented as percentages. The Chi-square test was applied to assess differences between categorical variables, with a *p*-value below 0.05 indicating statistical significance.

The bold values indicate total of positive and negative responses.

Table 4 also indicated that, regarding communication among the surgical team, 72.7% (*n* = 109) of nurses gave overall positive feedback, with the highest satisfaction again reported at Al Shifa Medical Complex (76.8%), followed by Nasser Medical Complex (73.6%) and Gaza European Hospital (59.1%), though differences were not statistically significant (*p*-value = 0.279). Notably, 72.0% of nurses believed communication between surgical team members was excellent, 74.0% said information was exchanged respectfully and professionally, while only 46.0% reported that the team cooperated well during patient positioning.

Regarding policies, procedures, protocols, and training, 66.7% (*n* = 100) of nurses provided overall positive responses, with Al Shifa Medical Complex leading at 73.2%, followed by Nasser Medical Complex (65.3%) and Gaza European Hospital (54.5%), though these differences were not statistically significant (*p*-vale = 0.273). A majority of nurses (64.0%) confirmed the use of the surgical safety checklist, while 81.3% reported that hospital administration provides policies and protocols to enhance intraoperative patient safety. Additionally, 82.7% stated that a monitoring program exists to oversee the application of protocols, and 73.3% noted that a patient safety protocol is available in the operating room. About 65.3% believed that electronic health information systems help improve protocol implementation. In terms of training, 68.7% had received some form of on-the-job patient safety training, 75.3% had training on protocol application, and 72.7% expressed a need for further training in patient safety as shown in Table 5.

**Table 5:** Responses of the study participants for policy, procedure, protocols and training; and adverse events by hospitals.

Variables	Total (*n* = 150)	Al Shifa medical complex (*n* = 56)	Nasser medical complex (*n* = 72)	European Gaza hospital (*n* = 22)	P. value
	No (%)	No (%)	No (%)	No (%)	
Policy, procedure, protocols and training					
1. Is the surgical safety checklist applied in your hospital?					
Yes	96 (64.0)	33 (58.9)	46 (63.9)	17 (77.3)	0.315
No	54 (36.0)	23 (41.1)	26 (36.1)	5.0 (22.7)	
2. Is the hospital administration providing polices and protocols which improve patient safety intra operative?					
Yes	122 (81.3)	47 (83.9)	59 (81.9)	16 (72.7)	0.512
No	28 (18.7)	9.0 (16.1)	13 (18.1)	6.0 (27.3)	
3. Is the hospital have monitoring program to follow up applications of protocols?					
Yes	124 (82.7)	49 (87.5)	59 (81.9)	16 (72.7)	0.293
No	26 (17.3)	7.0 (12.5)	13 (18.1)	6.0 (27.3)	
4. Is the patient safety protocol available at operation room department?					
Yes	110 (73.3)	39 (69.6)	53 (73.6)	18 (81.8)	0.548
No	40 (26.7)	17 (30.4)	19 (26.4)	4.0 (18.2)	
5. You think the electronic program (health information system) improving applications of patient safety protocol?					
Yes	98 (65.3)	39 (69.6)	46 (63.9)	13 (59.1)	0.636
No	52 (34.7)	17 (30.4)	26 (36.1)	9.0 (40.9)	
6. You have any course on job training intra operative about patient safety?					
Yes	103 (68.7)	40 (71.4)	48 (66.7)	15 (68.2)	0.846
No	47 (31.3)	16 (28.6)	24 (33.3)	7.0 (31.8)	
7. You believe, you need more training course about patient safety?					
Yes	109 (72.7)	43 (76.8)	52 (72.2)	14 (63.6)	0.499
No	41 (27.3)	13 (23.2)	20 (27.8)	8.0 (36.4)	
8. You have any training course about applications of protocols?					
Yes	113 (75.3)	42 (75.0)	53 (73.6)	18 (81.8)	0.735
No	37 (24.7)	14 (25.0)	19 (26.4)	4.0 (18.2)	
Total of positive responses	**100** (**66.7)**	**41** (**73.2)**	**47** (**65.3)**	**12** (**54.5)**	**0**.**273**
Total of negative responses	**50** (**33.3)**	**15** (**26.8)**	**25** (**34.7)**	**10** (**45.5)**	
Adverse events					
1. Was there any adverse anaesthesia event?					
Yes	113 (75.3)	38 (67.9)	55 (76.4)	20 (90.9)	0.076
No	37 (24.7)	18 (32.1)	17 (23.6)	2.0 (9.1)	
2. Was there any anaesthesia related mortality rate?					
Yes	127 (84.7)	46 (82.1)	62 (86.1)	19 (86.4)	0.803
No	23 (15.3)	10 (17.9)	10 (13.9)	3.0 (13.6)	
3. Unplanned return to operation room (for re-exploration, or any other reason which was otherwise not planned)?					
Yes	111 (74.0)	40 (71.4)	53 (73.6)	18 (81.8)	0.639
No	39 (26.0)	16 (28.6)	19 (26.4)	4.0 (18.2)	
4. Surgical site infection reported within 7 days post operatively?					
Yes	116 (77.3)	44 (78.6)	56 (77.8)	16 (72.7)	0.851
No	34 (22.7)	12 (21.4)	16 (22.2)	6.0 (27.3)	
5. The health indicators regarding patient safety not available?					
Yes	53 (35.3)	19 (33.9)	26 (36.1)	8.0 (36.4)	0.962
No	97 (64.7)	37 (66.1)	46 (63.9)	14 (63.6)	
6. Do not document medical mistake freely during surgery?					
Yes	102 (68.0)	40 (71.4)	49 (68.1)	13 (59.1)	0.575
No	48 (32.0)	16 (28.6)	23 (31.9)	9.0 (40.9)	
7. Your supervisor intended to document your professional mistake for punishment?					
Yes	113 (75.3)	41 (73.2)	55 (76.4)	17 (77.3)	0.894
No	37 (24.7)	15 (26.8)	17 (23.6)	5.0 (22.7)	
Total of positive responses	**140** (**93.3)**	**52** (**92.9)**	**67** (**93.1)**	**21** (**95.5)**	**0**.**910**
Total of negative responses	**10** (**6.7)**	**4.0** (**7.1)**	**5.0** (**6.9)**	**1.0** (**4.5)**	

Categorical variables were presented as percentages. The Chi-square test was applied to assess differences between categorical variables, with a *p*-value below 0.05 indicating statistical significance.

The bold values indicate total of positive and negative responses.

Finally, a significant majority of nurses (93.3%, *n* = 140) across the surveyed hospitals reported a high incidence of adverse events, with Gaza European Hospital having the highest rate (95.5%), followed by Nasser Medical Complex (93.1%) and Al Shifa Medical Complex (92.9%). However, these differences were not statistically significant (*p* = 0.910). Additionally, 75.3% of nurses observed adverse anesthesia events, while 84.7% reported anesthesia-related mortality. About 74.0% noted unplanned returns to the operating room, and 77.3% reported surgical site infections occurring within seven days post-surgery. Furthermore, 35.3% indicated the absence of patient safety health indicators, 68.0% said they were reluctant to document surgical errors, and 75.3% stated that their supervisors intended to record professional mistakes for punitive purposes as shown in Table 5.

## Discussion

This study aimed to assess nurses’ compliance with patient safety in operating rooms of governmental hospitals in the Gaza Strip, focusing on infection control, safety measures, communication, adherence to protocols, and adverse events. The findings provide important insights into the current state of perioperative safety practices in a resource-constrained and conflict-affected setting. The study revealed moderate compliance with sterilization and cleaning protocols, with less than half (46.7%) of nurses across hospitals reporting positive adherence to these practices. This rate, though concerning, aligns with findings from similar low-resource settings where infection control faces challenges due to limited supplies and infrastructure (16, 17). Al Shifa Medical Complex showed the highest compliance, possibly reflecting better resource availability or management, consistent with findings of previous study (18).

Intraoperative precautions were followed by approximately 73.3% of nurses, with proper hand scrubbing and accurate recording of incision times receiving relatively high compliance rates. However, gaps were evident in checklist adherence and operative note documentation, echoing studies in Jordan and Egypt where incomplete checklist use and documentation were linked to increased surgical risks (11, 12). These findings underscore the need for continuous staff training and reinforcement of protocol compliance to enhance surgical safety. Regarding immediate post-operative monitoring, 68.7% adherence suggests moderate vigilance in recovery care. Higher compliance with oxygen saturation monitoring and pain assessment indicates awareness of critical post-op parameters, similar to observations in other low-middle income countries settings (19). Yet, variability among hospitals and lower compliance in some monitoring aspects suggest resource or staffing limitations that require addressing. Communication among surgical teams was perceived positively by nearly three-quarters of nurses, although cooperation during patient positioning was less favorably viewed. Effective communication is a cornerstone of patient safety, and these findings parallel global literature emphasizing teamwork as crucial for reducing errors (20). The slight shortfall in cooperation highlights an area for targeted team-building and communication training. Policies, procedures, protocols, and training were reported positively by around two-thirds of nurses. While the surgical safety checklist was reportedly in use by 64% of nurses, this is lower than the near-universal adoption rates in some high-income countries (21). Training gaps were also identified, with over 70% expressing a need for further patient safety education, reinforcing the call for ongoing professional development. Furthermore, the study highlighted a high reported incidence of adverse events, with over 90% of nurses acknowledging frequent complications such as anesthesia-related issues and surgical site infections. The reluctance to document errors due to fear of punitive measures echoes findings worldwide that blame culture remains a significant barrier to transparent reporting and learning (22). This culture hinders safety improvements and necessitates systemic change.

The moderate level of adherence to infection control and safety practices observed in Gaza's governmental hospitals highlights the impact of challenging working environments and limited resources. The incomplete application of safety protocols likely plays a role in the high number of adverse events reported by nursing staff. Although communication and teamwork among surgical teams are generally viewed in a positive light, they still require targeted improvement to ensure seamless coordination throughout all stages of the perioperative process. The notable demand for training among nurses underscores a pressing need to enhance their competencies in patient safety. Additionally, the prevailing punitive culture associated with error reporting presents a barrier to open dialogue and learning. Addressing this issue is critical for fostering an environment where staff feel safe to report incidents without fear of retribution. This can be achieved through the implementation of non-punitive reporting systems, such as anonymous submissions and constructive, educational feedback. To advance patient safety in operating rooms, continuous and structured training programs focusing on infection prevention, protocol compliance, and communication skills must be implemented, with regular refresher courses to maintain consistent standards. Ensuring the availability of essential resources including sterilization equipment, protective gear, and proper monitoring tools, as well as improving and maintaining surgical infrastructure, is also vital. Enforcing the mandatory use of standardized tools like the WHO Surgical Safety Checklist through ongoing supervision and regular audits can help improve procedural consistency and accountability. Furthermore, promoting teamwork and communication through interprofessional training workshops can strengthen collaboration and enhance information sharing among surgical staff. Lastly, the establishment of effective monitoring and evaluation mechanisms to track safety metrics and adverse events will support continuous quality improvement and evidence-based decision-making in surgical care. The current study clearly demonstrates that nurses understand the importance of infection control and safety protocols but face only moderate levels of compliance in practice. Communication and teamwork were identified as key areas requiring improvement. Their perspectives emphasize the need for improved training, increased resources, and a supportive, non-punitive atmosphere to enhance patient safety in the operating rooms of Gaza.

### Strength and limitations

This study's main strength is its broad inclusion of multiple government hospitals in the Gaza Strip, offering a wide perspective on nurses’ views of patient safety in operating rooms. Its large sample size and high response rate strengthen the reliability and applicability of the results in similar low-resource environments. However, the use of self-reported data may introduce biases like social desirability and recall errors, potentially affecting response accuracy. Additionally, the cross-sectional design restricts causal conclusions and the ability to observe changes over time. Future research should use mixed methods, such as direct observation, longitudinal monitoring of adverse events, and qualitative approaches, to better explore cultural and systemic factors impacting patient safety.

## Conclusion

This study provides valuable insights into nurses’ perceptions of patient safety in the operating rooms of Gaza Strip governmental hospitals. While adherence to key infection control and safety measures is moderate, significant gaps persist, particularly in protocol adherence, documentation, and adverse event reporting. The findings highlight the urgent need for enhanced training, resource support, and a cultural shift toward non-punitive error reporting to improve patient safety outcomes. Policymakers and hospital leaders must prioritize these areas to strengthen surgical safety in this challenging context, ultimately improving care quality and patient wellbeing.

## Data Availability

The raw data supporting the conclusions of this article will be made available by the authors, without undue reservation.
